# Characteristics of mitral valve leaflet length in patients with pectus excavatum: A single center cross-sectional study

**DOI:** 10.1371/journal.pone.0212165

**Published:** 2019-02-11

**Authors:** Koutatsu Nomura, Yoichi Ajiro, Satomi Nakano, Maiko Matsushima, Yuki Yamaguchi, Nahoko Hatakeyama, Mari Ohata, Miyuki Sakuma, Terumi Nonaka, Miyuki Harii, Masafumi Utsumi, Kazuhiro Sakamoto, Kazunori Iwade, Nobuo Kuninaka

**Affiliations:** 1 Department of Clinical Laboratory, National Hospital Organization Yokohama Medical Center, Yokohama, Kanagawa, Japan; 2 Department of Cardiology, National Hospital Organization Yokohama Medical Center, Yokohama, Kanagawa, Japan; 3 Department of Cardiology, Tokyo Women’s Medical University, Shinjuku, Tokyo, Japan; 4 Department of Respiratory Surgery, National Hospital Organization Yokohama Medical Center, Yokohama, Kanagawa, Japan; Imperial College, London, UK, UNITED KINGDOM

## Abstract

The mitral valve morphology in patients with pectus excavatum (PE) has not been fully investigated. Thirty-five patients with PE, 46 normal controls, and patients with hypertrophic cardiomyopathy (HCM) who underwent 2 leaflet length measurements of Carpentier classification P2 and A2 using a transthoracic echocardiography were retrospectively investigated. The coaptation lengths and depths, papillary muscle tethering length, and mitral annular diameters were also measured. The P2 and A2 lengths were separately compared between 2 groups: older than 16 years and 16 years or younger. Furthermore, the correlations between actual P2 or A2 lengths and Haller computed tomography index, an index of chest deformity, were investigated in patients with PE exclusively. Among subjects older than 16 years, patients with PE had significantly shorter P2, longer A2, shorter copatation depth, and longer papillary muscle tethering length compared with normal controls. Similarly, patients with PE had significantly shorter P2 and shorter coaptation depth even compared with patients with HCM, while no significant difference was found in A2 length and papillary muscle tethering length. The same tendency was noted between 4 normal controls and 7 age- and sex-matched patients with PE ≤ 16 years old. No significant difference regarding A2/P2 ratio was found between patients with PE older and younger than 16 years. No significant correlation between the Haller computed tomography index and actual mitral leaflet lengths in patients with PE older than 16 years was noted; the same was observed for A2/P2 in all patients with PE. In conclusion, the characteristic features of the shorter posterior mitral leaflet, the longer anterior mitral leaflet, the shorter coaptation depth, and the longer papillary muscle tethering length in patients with PE was demonstrated. This finding might provide a clue regarding the etiology of mitral valve prolapse in PE at its possible earliest form.

## Introduction

Pectus excavatum (PE) is a congenital deformity in which the anterior chest sinks like a funnel around the xiphoid process [1,2]. Pectus excavatum can be accompanied by heritable connective tissue disease and many cardiovascular complications, including mitral valve prolapse (MVP). Because the cardiovascular complications are important in PE for predicting prognosis and for considering surgical indications, evaluating mitral valve morphology is important for patients with PE.

Herein, we conduct a retrospective study to assess the mitral valve morphology in patients with PE using the mitral leaflet length as measured by transthoracic echocardiography.

## Materials and methods

### Study design and population

**Fig 1:**
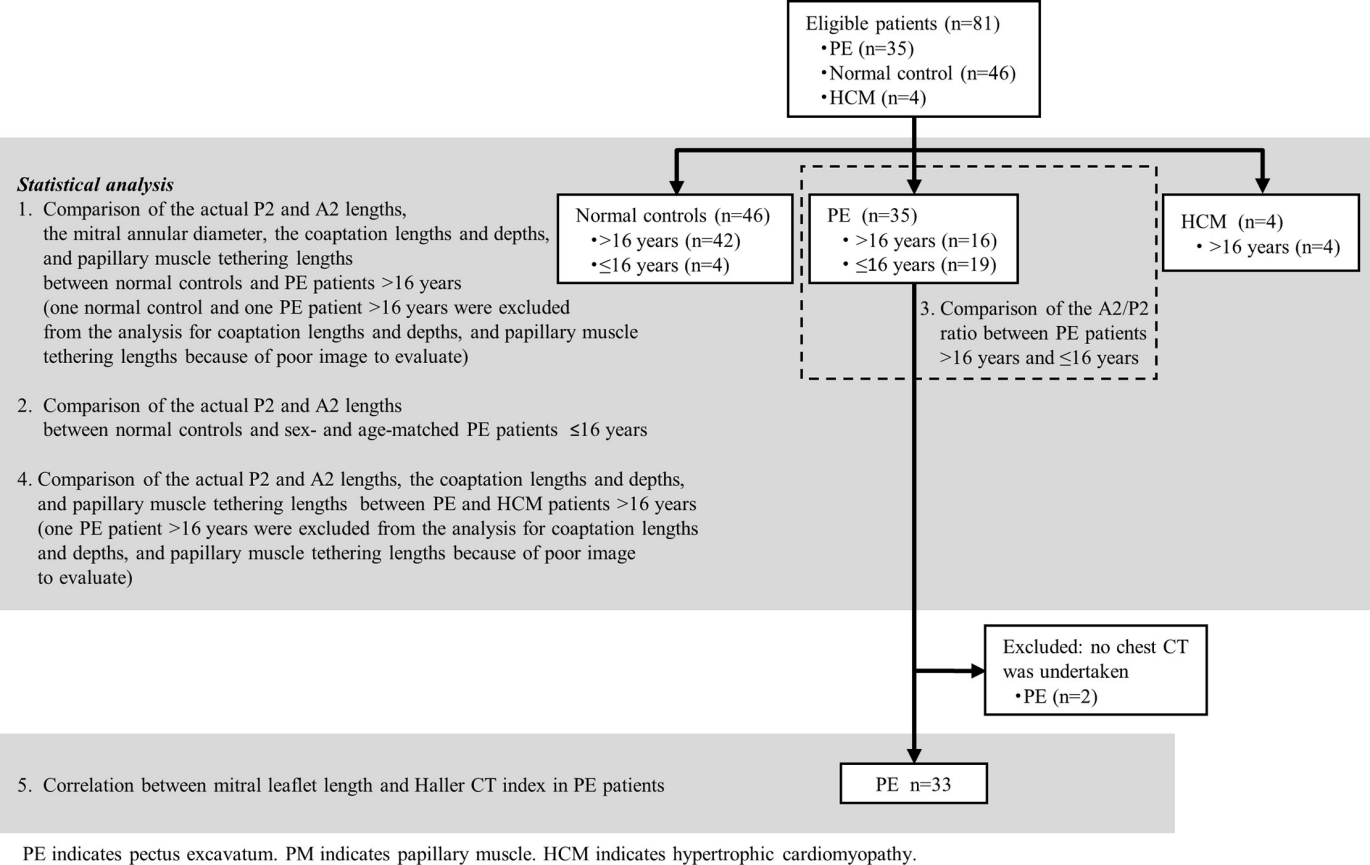
Flow chart of the study.

Thirty-five consecutive patients who were diagnosed with PE and who underwent transthoracic echocardiography between November 2012 and March 2014 were registered in this single-center retrospective cross-sectional study. Moreover, 46 healthy individuals with no history of heart failure, atrial fibrillation, myocardial infarction, cardiomyopathy, pericardial disease, or valvular heart disease from the same period were registered as normal controls. Patients with PE and normal controls were excluded if 2 mitral leaflets, Carpentier classification P2 (hereafter P2) and A2 (hereafter A2), could not be measured (Fig 1). In addition to normal controls, the four patients with familial or sarcomere related mutation-positive hypertrophic cardiomyopathies were also registered because hypertrophic cardiomyopathy had genetic basis and mechanically distorted force to mitral valve [3]. This retrospective cross sectional study was conducted by an opt-out methods in which study information was announced on hospital homepage and gave all participants or their guardians an opportunity to disagree with using their medical records. Neither written nor verbal consent were obtained. This study was registered in the University Hospital Medical Information Network Clinical Trials Registry (identification number: UMIN000029508; URL: http://www.umin.ac.jp/ctr/index.htm) and conducted under the review and approval of the Yokohama Medical Center Review Board (approval reference no. 29–2; URL: http://www.yokohama-mc.jp/research/rinri.html).

### Echocardiographic measurements

**Fig 2:**
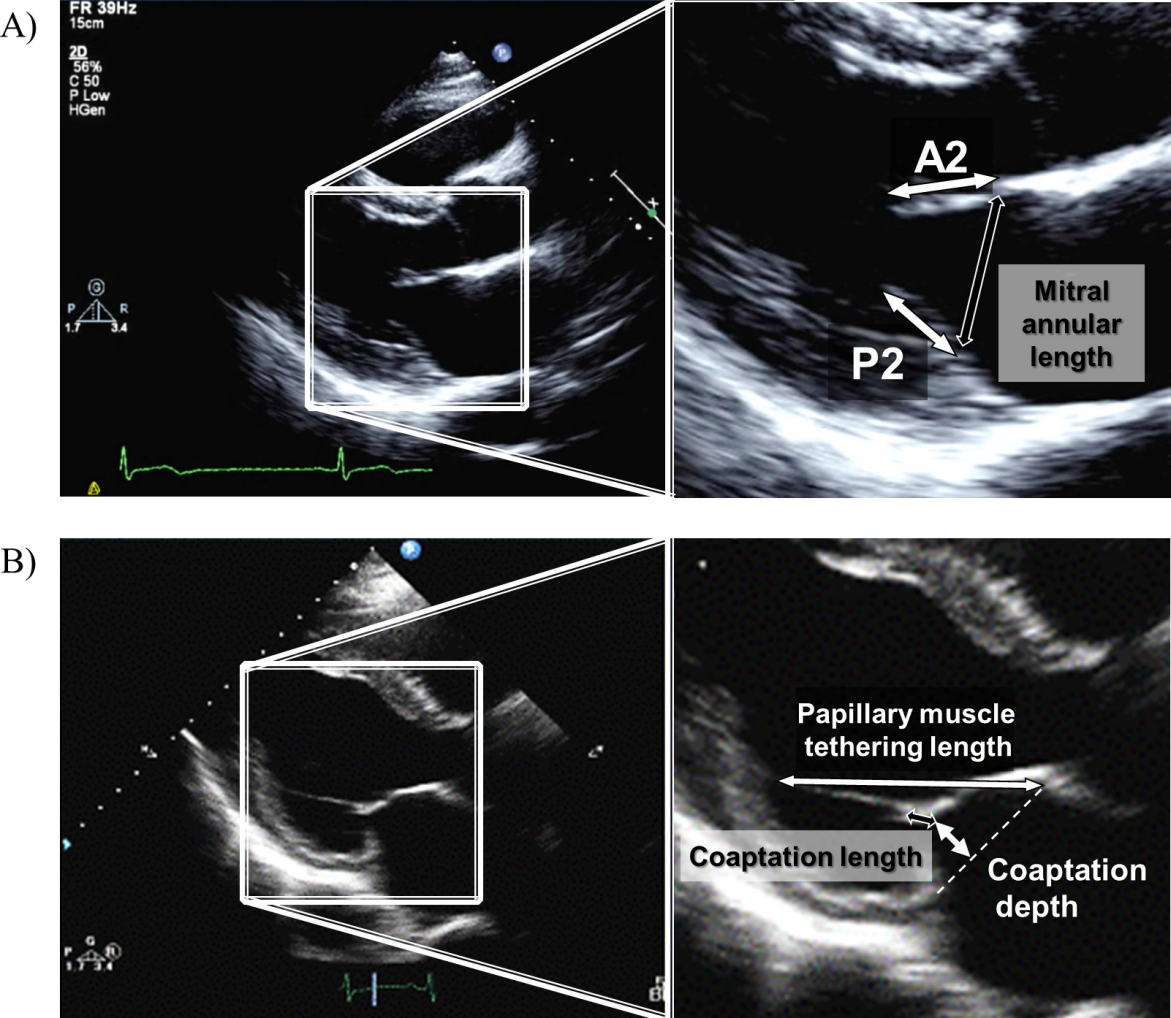
Measurement of mitral leaflet lengths, mitral annular lengths, coaptation lengths and depths, and papillary muscle tethering lengths. (A) The P2 (white bidirectional arrow) and A2 (white bidirectional arrow) lengths, and mitral annular lengths (black bidirectional arrow) were measured at end-diastole using a left ventricular long axis view. (B) The coaptation lengths (white bidirectional arrow) and depths (black bidirectional arrow), and papillary muscle tethering lengths (white bidirectional arrow) were measured at end-systole using a left ventricular long axis view.

Measurements of P2 and A2 leaflet lengths were performed by iE33 (Philips Electronic Japan, Tokyo, Japan) with an ultrasonic probe S5-1 (transmitted waveform frequency, 1–5 MHz; Philips Electronic Japan) or by HD15 (Philips Electronic Japan) with an ultrasonic probe S5-2 (transmitted waveform frequency, 1–4 MHz; Philips Electronic Japan). P2 and A2 lengths, and mitral annular diameters were measured at end-diastole using a left ventricular long axis view (Fig 2A) by 2 independent echocardiologists. The coaptation lenths and depths, and papillary muscle tethering lengths were measured at end-systole using a left ventricular long axis view (Fig 2B) by 2 independent echocardiologists. Mitral valve prolapse was defined as the superior displacement of one or both mitral leaflets into the left atrium exceeding more than 2 mm from the mitral annulus during the systolic phase, using a left ventricular long axis view [4].

### Computed tomographic measurements

**Fig 3:**
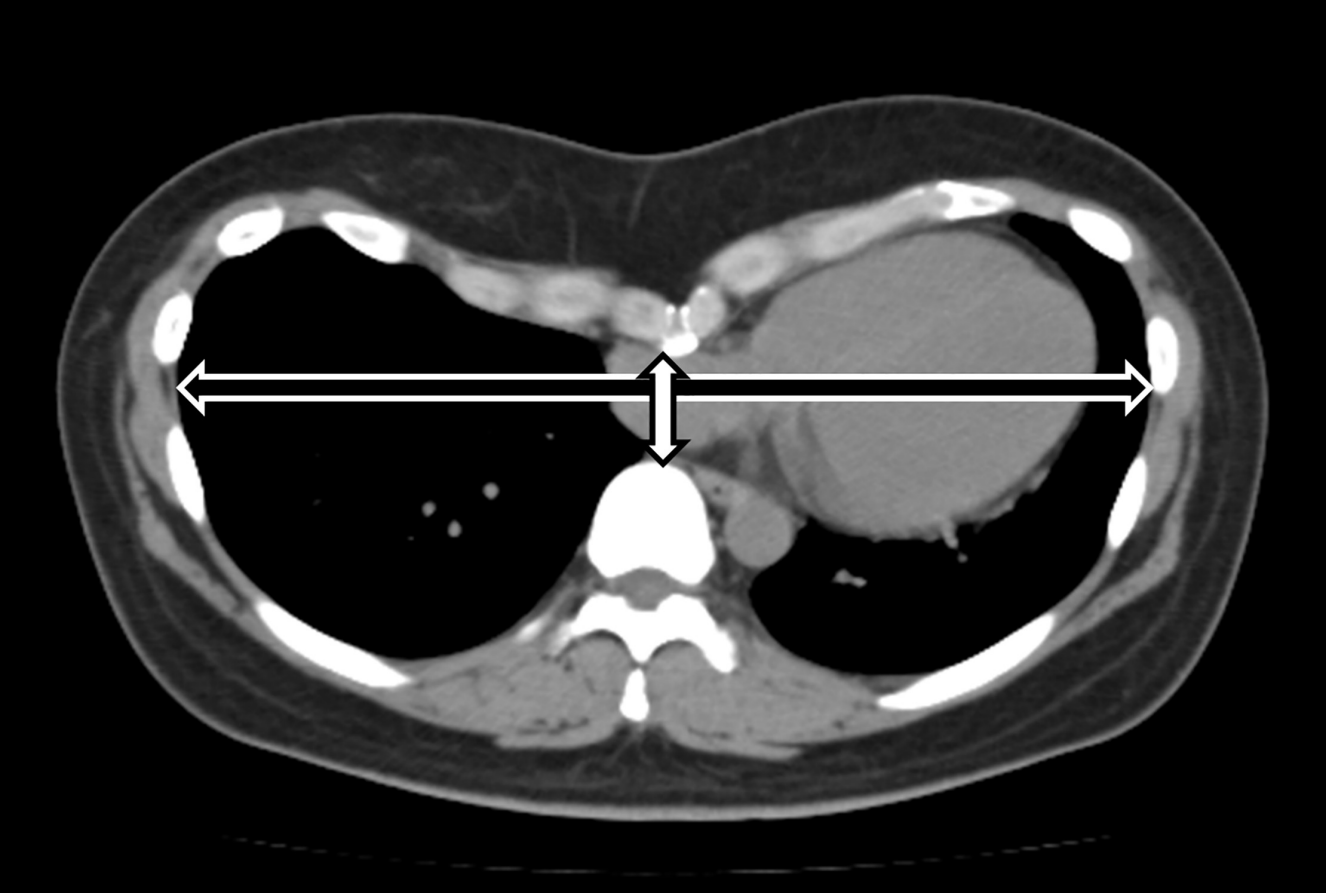
Measurement of the Haller computed tomography (CT) index. The Haller CT index was calculated by dividing the thoracic transverse diameter (black bidirectional arrow) by the distance between the sternum and the vertebra (white bidirectional arrow).

The extent of chest deformity in patients with PE was estimated using the Haller computed tomography (CT) index, in which the transverse diameter of the thorax was divided by the diameter between the sternum and the vertebrae during chest CT [5] (Fig 3).

### Statistical analysis

Since there was no established standardized method for the measurement of the mitral leaflet length during the growth period, we assessed the characteristics of the mitral leaflet length in PE as follows: (1) the actual P2 and A2 leaflet lengths, and mitral annular diameters between normal controls and patients with PE older than 16 years were compared. Similarly, the coaptation lengths and depths, and papillary muscle tethering lengths between normal controls and patients with PE older than 16 years whose end-systolic long axis view were sufficient quality for reliable measurement were compared; (2) the actual P2 and A2 leaflet lengths between 4 normal controls and 7 age- and sex-matched patients with PE 16 years or younger were compared individually (no statistical analysis was conducted due to the small sample number); (3) the A2/P2 ratio, which was calculated to reduce the influence of the growth period, between patients with PE older than 16 years and those 16 years or younger was compared; (4) the actual P2 and A2 leaflet lengths, coaptation length, coaptation depth, and papillary muscle tethering length between the patients with PE and with HCM were compared; and (5) to evaluate the correlation between the extent of chest deformity and the mitral leaflet lengths, the Spearman rank correlation coefficients between the Haller CT index and the actual P2 and A2 mitral leaflet lengths in patients with PE older than 16 years and between the Haller CT index and the A2/P2 ratio in all patients with PE were calculated. The deviation of patient characteristics among normal controls, PE patients, and HCM patients was analyzed by one-way ANOVA test for continuous valuables and by χ^2^ test for categorical valuables. Comparisons between patients with PE and normal controls were conducted using the Student’s t-test for continuous valuables and χ^2^ test for categorical valuables. In the case of non-normal distribution continuous variables, logarithmically converted valuables were used for analysis. A probability value (hereafter p value) <0.05 was considered statistically significant. Continuous variables are presented as mean ± standard deviation. Categorical valuables are presented as number and percentage.

All statistical analyses were performed with JMP software, version 12.0 (SAS Institute Japan Co. Ltd., Tokyo, Japan). The validity of the statistical methods and their results was confirmed by a statistics expert.

## Results

### Characteristics of study patients

Thirty-five patients with PE (16 patients older than 16 years; range, 3–70 years), 46 control subjects (42 control subjects older than 16 years; range, 8–84 years), and 4 patients with HCM (2 familial HCMs and 2 sarcomere related mutation-positive HCMs older than 16 years; range, 67–76 years) whose P2 and A2 mitral valve leaflets could be measured, were registered in the present study (Fig 1).

The characteristics of the patients older than 16 years are shown in Table 1. No statistically significant deviations in indices, including sex, height, weight, and body surface area, were found among patients with PE, normal controls, and patients with HCM older than 16 years. Meanwhile, significant deviations were noted in all above-mentioned indices, except sex (S1 Table), in all patients, including those 16 years or younger.

**Table 1:** Patient characteristics of normal controls and patients with PE older than 16 years.

	Normal controls (n = 42)	PE (n = 16)	HCM (n = 4)	p value
Age, years	58.8 ± 15.3	31.6 ± 13.8	72.3 ± 13.8	<0.0001
Males	26 (61.9)	9 (56.3)	2 (50.0)	0.8520
Height, m	1.63 ± 0.09	1.68 ± 0.09	1.57 ± 0.09	0.0665
Body weight, kg	59.1 ± 14.4	56.5 ± 12.1	48.7 ± 6.8	0.3246
Body surface area, m^2^	1.63 ± 0.23	1.63 ± 0.20	1.50 ± 0.11	0.3194
Mitral valve prolapse	0 (0.0)	1 (8.3)	0 (0.0)	0.2320

Values are presented as ± standard deviation or n (%). HCM indicates hypertrophic cardiomyopathy. PE indicates pectus excavatum.

### Comparison of the mitral leaflet lengths, mitral annular diameters, coaptation lengths and depths, and papillary muscle tethering lengths of normal controls and PE patients

**Fig 4:**
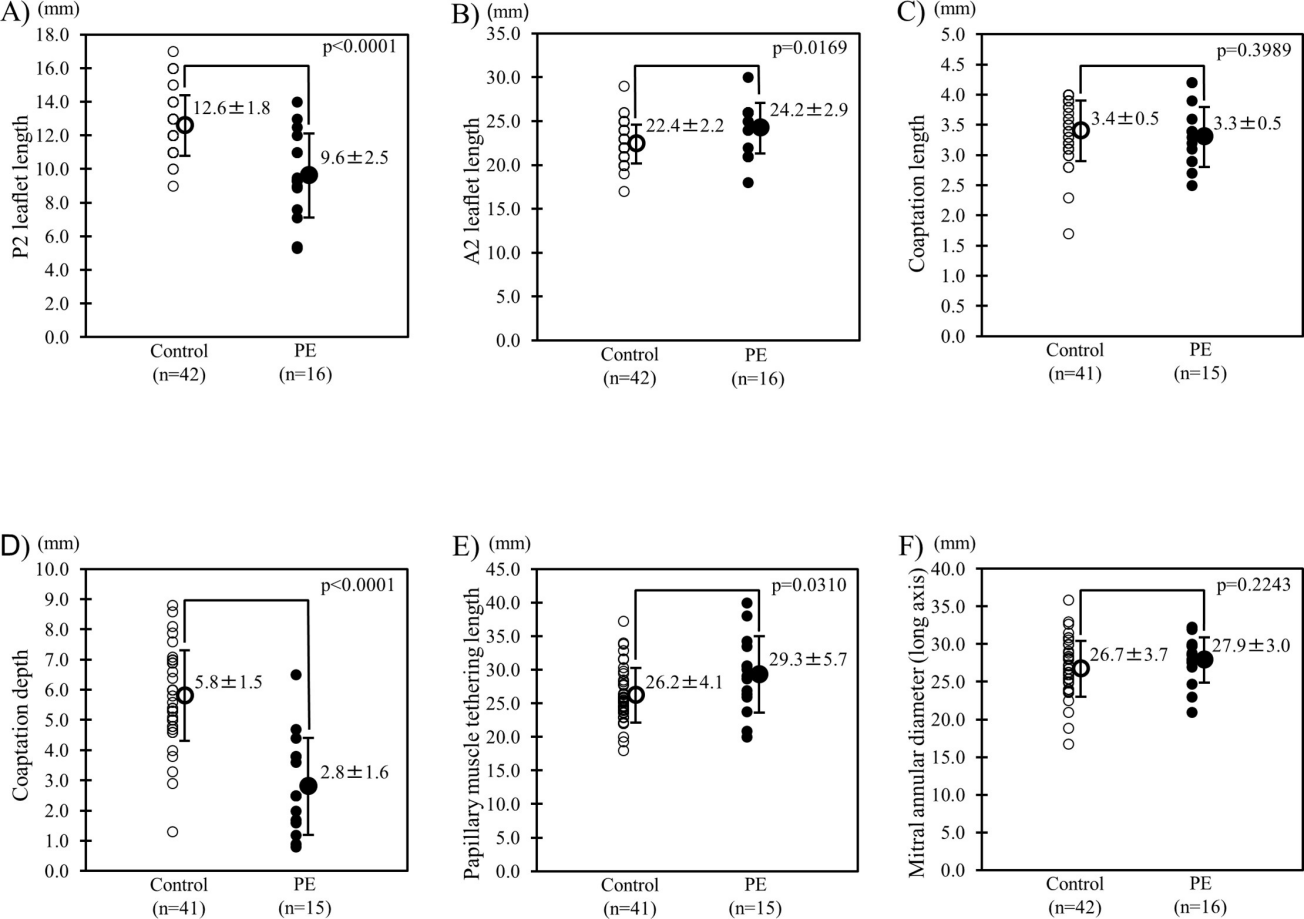
Comparison of the mitral leaflet length, coaptation length and depth, and papillary muscle tethering length, and mitral annular diameter between normal controls and patients with pectus excavatum (PE). Comparison of the actual P2 (A), A2 length (B), coaptation length (C) and depth (D), papillary muscle tethering length (E), and mitral annular diameter (F) between normal controls and patients with PE older than 16 years.

**Fig 5:**
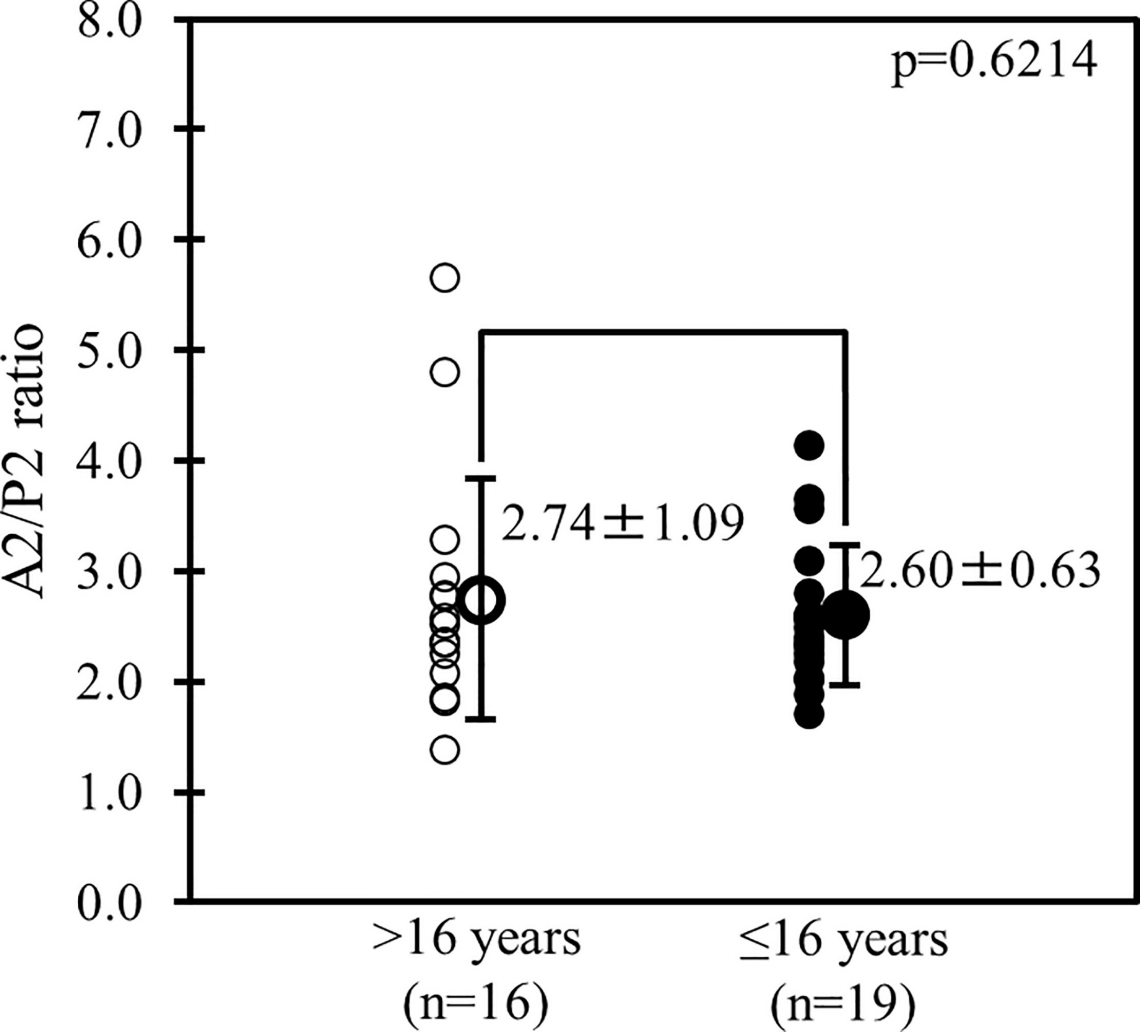
Comparison of the A2/P2 ratio in patients with pectus excavatum (PE). Comparison of the A2/P2 ratio between patients with PE older than 16 years and those 16 years or younger.

Among patients older than 16 years, patients with PE had significantly shorter P2, longer A2, shorter coaptation depths, and longer papillary muscle tethering lengths when compared with normal controls while no statistically significance was found in mitral annular diameters and coaptation lengths (Fig 4). Meanwhile, patients with PE, who were 16 years or younger, seemed to have similar characteristics compared with normal controls (Table 2). Among the 35 patients with PE, no statistical significance was detected regarding the A2/P2 ratio between those older than 16 years and those 16 years or younger (Fig 5).

**Table 2:** Comparison of the actual P2 and A2 lengths, coaptation lengths and depths, papillary muscle tethering lengths, and mitral annular diameters between normal controls and sex- and age-matched patients with PE younger than 16 years.

Patient			Leaflet length		Coaptation		PM tethering	Mitral annular
no age, gender disease			P2	A2	length	depth	length	diameter
			(mm)	(mm)	(mm)	(mm)	(mm)	(mm)
#1	8, male	Normal control	12	22	1.8	3.6	14.2	22
#2		PE	5.6	20	5.5	2.1	18.5	22
#3	12, female	Normal control	8.1	18	3.3	3.0	19.5	20
#4		PE	6.9	18	4.6	2.6	29.6	19
#5	13, male	Normal control	12	23	4.5	2.5	25.3	27
#6		PE	9.8	23	5.5	0.6	27.7	25
#7	15, male	Normal control	14	23	2.2	4.0	21.3	24
#8		PE	10	24	3.2	3.5	34.1	30
#9		PE	11	24	1.7	0.9	23.9	28
#10		PE	9.3	24	3.5	2.7	25.8	22
#11		PE	7.0	29	4.3	1.7	26.0	28

PE indicates pectus excavatum. PM indicates papillary muscle.

### Comparison of the mitral leaflet lengths of PE and HCM patients

**Fig 6:**
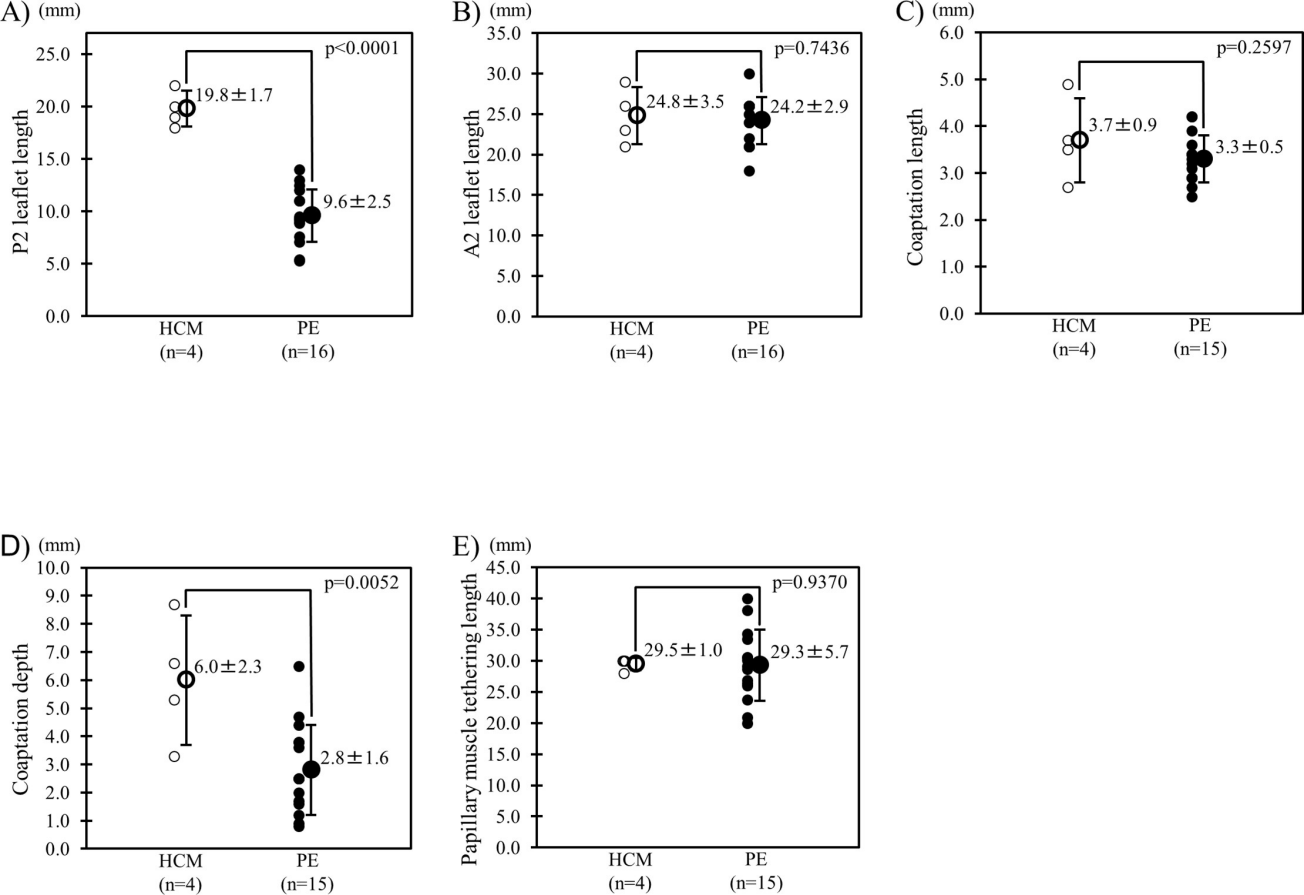
Comparison of the mitral leaflet length, coaptation length and depth, and papillary muscle tethering length betwee the patients with PE and HCM. Comparison of the actual P2 (A), A2 length (B), coaptation length (C) and depth (D), and papillary muscle tethering length (E) between the patients with PE and HCM older than 16 years.

Among PE and HCM patients older than 16 years, PE patients had significantly shorter P2 and coaptation depth while no significant difference in A2 length, coaptation length, and papillary muscle tethering length when compared with HCM patients (Fig 6).

### Correlation to Haller CT index

**Fig 7:**
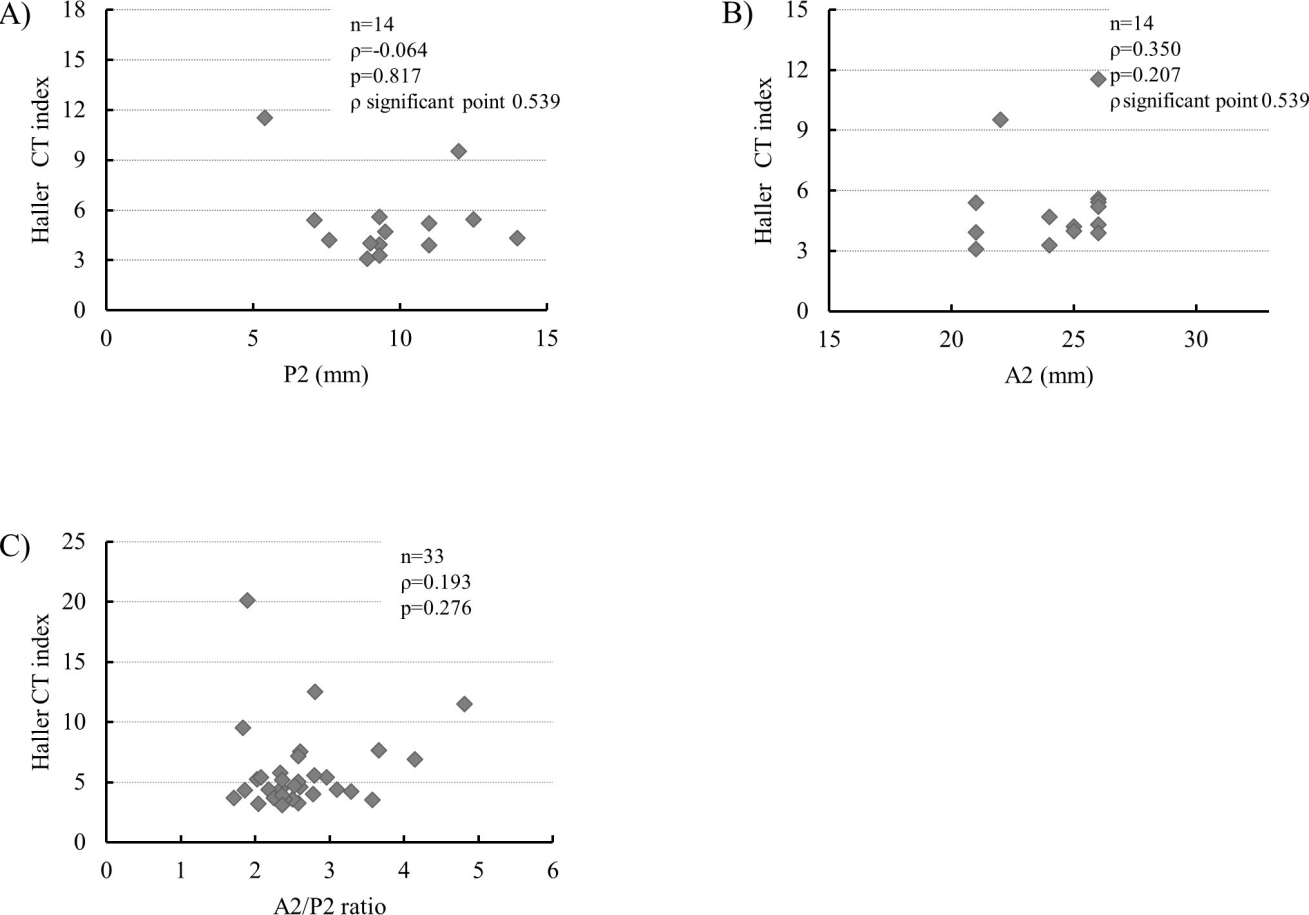
Correlation between mitral leaflet length and Haller CT index in patients with pectus excavatum (PE). P2 (A) and A2 lengths (B) of patients with PE older than 16 years. (C) A2/P2 ratio of all patients with PE.

Among the 35 patients with PE, 33 patients who underwent chest CT were analyzed (Fig 1). No significant correlation was shown between the P2 and A2 actual lengths and the Haller CT index in the patients with PE older than 16 years (Fig 7A and 7B). Similarly, no significant correlation was shown between the A2/P2 ratio and the Haller CT index in all patients with PE (Fig 7C).

### Cardiovascular complications accompanying PE

A mitral anterior leaflet prolapse with mild regurgitation was noted in a 19-year-old female patient with PE (Table 1). Her P2 and A2 lengths were 5.4 and 26.0 mm, respectively, showing a shorter posterior and a longer anterior mitral leaflet compared with the mean P2 and A2 lengths in patients with PE older than 16 years in the present study. In addition, her Haller CT index was 11.5, which was higher compared with the mean Haller CT index of 5.8 in all patients with PE. Among patients with PE 16 years or younger, each individual presented with an atrial septal defect, an aortic bicuspid valve, and a Kawasaki disease, and two patients presented mild tricuspid regurgitation with floppy valve (S2 Table).

## Discussion

The present study demonstrated that (1) patients with PE have shorter posterior mitral leaflets and longer anterior mitral leaflets, shorter coaptation lengths, and longer papillary muscle tethering lengths compared with normal controls, (2) no statistically significant difference was found regarding the A2/P2 ratio between patients with PE older than 16 years and those 16 years or younger, and (3) no statistically significant correlation was found between the Haller CT index and the mitral leaflet lengths in patients with PE.

Mihăilă et al. [6] and Kalmanson [7] reported that the mitral leaflet lengths of healthy individuals were 10 ± 3 and 14 ± 0.9 mm, respectively, in the posterior leaflet and 22 ± 5 and 23 ± 0.9 mm, respectively, in the anterior leaflet. Those of normal controls in the present study were 12.6 ± 1.8 mm in P2 and 22.4 ± 2.2 mm in A2, which are similar to the previous reports mentioned. Moreover, P2 length (9.6 ± 2.5 mm) was shorter and A2 length (24.2 ± 2.9 mm) was longer in patients with PE in the present study compared with the leaflet lengths in these reports. These findings support the observation of a shorter posterior mitral leaflet and a longer anterior mitral leaflet in patients with PE.

The characteristics of mitral valve morphology in patients with PE were chiefly reported to be related to MVP syndrome [8–12]. Embryotic and/or hereditary disorders were suggested as the etiology [12–14]. The embryotic disorder hypothesis was based on the finding that both periods when the atrioventricular valve differentiates into the mitral valve and when the sternum and ribs start chondrifying and ossifying were around the 5th to the 6th week of gestation. Therefore, intrinsic and/or extrinsic stress during this period may possibly cause both thoracic and mitral valve malformations [1,13,14]. While the actual incidence of connective tissue disease in PE cohort is unclear [1,20], the hereditary disorder hypothesis was suggested in connection with connective tissue diseases, such as Marfan syndrome [12,13,15,16]. Notably, the mutations related to elastin, collagen, and proteoglycans–the components of the mitral valve substrates [17]–have been reported as the cause of connective tissue diseases [18–20]. In the Marfan syndrome model using fibrilin-1-deficient mice, generation of myxomatous mitral valve via excessive tissue growth factor β1 signaling was reported [21], and in a study of 34 PE lineages, the mutation was found to relate to fibrilin, collagen, and tissue growth factor in some PE lineages [22]. Together with the findings of these reports, the possibility that PE and mitral valve malformation could be derived from a common inherited aberration was suggested. On the other hand, possible contribution of mechanical distortion of heart in PE patients was suggested with regard to right-sided heart anomalies including tricuspid regurgitation [23]. The present study suggested both genetic predisposition and mechanical distortion as possible cause: (1) that the characteristic features of mitral valves, i.e., a shorter posterior leaflet and a longer anterior leaflet in PE, are probably present already during childhood suggested the possible involvement of genetic predisposition; and (2) that the consistency in finding the above-mentioned characteristic features–in other words, no longer posterior leaflet or shorter anterior leaflet–, a longer papillary muscle tethering length in PE patients, and no shorter posterior leaflet length in HCM patients suggested the possible involvement of mechanical distortion. Together with those findings leads to our hypothesis that the continuous mechanical stress by the thoracic deformity may have at least some role in generating mitral valve malformation regardless of whether there was a genetic predisposition.

Severe mitral regurgitation, a serious cardiac event of MVP that requires surgery, often manifests in the fifth and sixth decades of life in patients with MVP as valve degeneration, with accompanying leaflet regression and fibromyxomatous change with or without hereditary predisposition. It can be considered that the large mitral leaflet would suffer larger closing force and longer papillary muscle tethering lengths would exert larger tethering force both of which might promote leaflet and chordae degeneration, i.e. the myxomatous changes to the mitral valve. Together with the findings of short coaptation depths, following three consideration would arose: (1) even a minor tone chordae as a result of advanced degeneration would cause mitral prolapse and significant regurgitation; (2) the continuous mechanical stress would promote degeneration, shrink leaflet gradually, and would cause mitral prolapse and significant regurgitation as well; and (3) the characteristically shorter posterior mitral leaflet, longer anterior mitral leaflet, and accompanying shorter coaptation depth found in the present study might be an early possible form of MVP in PE. As Delling and Vasan [16] implied the importance of recognizing the early forms of MVP, we believe the findings of this study would be important in the pathogenesis and natural history of MVP in PE.

Because the present study has a single-center retrospective cross-sectional design, limitations include a small sample number and patient selection bias. There may be potential bias because of the selection of normal controls without matching to patients with PE. Moreover, the technical limitation due to unavailability of 3-dimensional echological measurement exists in the present study. It would be preferable to conduct a well-designed prospective cohort study with data retrieved via genetic examination to evaluate hereditary predisposition and acquired modifier influence.

## Conclusion

The present study demonstrated that a short posterior leaflet, a long anterior leaflet, a short coaptation depth, and a long papillary muscle tethering length of the mitral valve are the characteristic features of PE. This finding might provide a clue regarding the etiology of MVP in PE at its possible earliest form.

## Supporting information

S1 Table Patient characteristics of all normal controls and all patients with PE. Values are presented as ± standard deviation or n (%). PE indicates pectus excavatum. ^a^ n = 43.
(DOCX)

S2 Table Cardiovascular complications accompanying PE. ASD indicates atrial septal defect. TR indicates tricuspid regurgitation. AR indicates aortic regurgitation. MVP indicates mitral valve prolapse.
(DOCX)

## References

[1] Brochhausen C, Turial S, Müller FK, Schmitt VH, Coerdt W, Wihlm JM, et al Pectus excavatum: history, hypotheses and treatment options. Interact Cardiovasc Thorac Surg. 2012;14:801–6. 10.1093/icvts/ivs045 22394989 PMC3352718

[2] Bauhinus J. Observatio. In: Ioannis Schenckii a Grafenberg, ed. Johannes Observatorium Medicarum, Rararum, Novarum, Admirabilium, et Montrosarum, Liber Secundus. Frankfurt: De partibus vitalibus, thorace contentis; 1609. pp. 322.

[3] Petrone RK, Klues HG, Panza JA, Peterson EE, Maron BJ. Coexistence of mitral valve prolapse in a consecutive group of 528 patients with hypertrophic cardiomyopathy assessed with echocardiography. J Am coll Cardiol. 1992;20:55–61. 1607539 10.1016/0735-1097(92)90137-c

[4] Freed LA, Levy D, Levine RA, Larson MG, Evans JC, Fuller DL, et al Prevalence and clinical outcome of mitral-valve prolapse. N Engl J Med. 1999;341:1–7. 10.1056/NEJM199907013410101 10387935

[5] Haller JA Jr., Kramer SS, Lietman SA. Use of CT scans in selection of patients for pectus excavatum surgery: a preliminary report. J Pediatr Surg. 1987;22:904–6. 3681619 10.1016/s0022-3468(87)80585-7

[6] Mihăilă S, Muraru D, Piasentini E, Miglioranza MH, Peluso D, Cucchini U, et al Quantitative analysis of mitral annular geometry and function in healthy volunteers using transthoracic three-dimensional echocardiography. J Am Soc Echocardiogr. 2014;27:846–57. 10.1016/j.echo.2014.04.017 24891260

[7] Kalmanson D. The mitral valve: a pluridisciplinary approach: Pub. Sciences Group; 1976.

[8] Saint-Mezard G, Duret JC, Chanudet X, Larrue J, Bonnet J, Bricaud H. Mitral valve prolapse and pectus excavatum. Fortuitous association or syndrome? Presse Med. 1986;15:439. 2938173

[9] Seliem MA, Duffy CE, Gidding SS, Berdusis K, Benson DW Jr. Echocardiographic evaluation of the aortic root and mitral valve in children and adolescents with isolated pectus excavatum: comparison with Marfan patients. Pediatr Cardiol. 1992;13:20–3. 10.1007/BF00788224 1736263

[10] Kusaka Y, Fukuda N, Asai M, Tominaga T, Ohshima C, Yamamoto M, et al Phono- and echocardiographic studies of the genesis of mitral valve prolapse in patients with funnel chest. J Cardiogr. 1984;14:731–41. 6543872

[11] Park JM, Varma SK. Pectus excavatum in children: diagnostic significance for mitral valve prolapse. Indian J Pediatr. 1990;57:219–22. 2246020 10.1007/BF02722092

[12] Udoshi MB, Shah A, Fisher VJ, Dolgin M. Incidence of mitral valve prolapse in subjects with thoracic skeletal abnormalities: a prospective study. Am Heart J. 1979;97:303–11. 420069 10.1016/0002-8703(79)90429-0

[13] Bon Tempo CP, Ronan JA Jr., de Leon AC Jr., Twigg HL. Radiographic appearance of the thorax in systolic click-late systolic murmur syndrome. Am J Cardiol. 1975;36:27–31. 1146694 10.1016/0002-9149(75)90863-2

[14] David J. Human developmental anatomy. New York: Ronald Press; 1963.

[15] Dean JC. Marfan syndrome: clinical diagnosis and management. Eur J Hum Genet. 2007;15:724–33. 10.1038/sj.ejhg.5201851 17487218

[16] Gould RA, Sinha R, Aziz H, Rouf R, Dietz HC 3rd, Judge DP, et al Multi-scale biomechanical remodeling in aging and genetic mutant murine mitral valve leaflets: insights into Marfan syndrome. PLoS One. 2012;7: e44639 10.1371/journal.pone.0044639 22984535 PMC3439411

[17] Delling FN, Vasan RS. Epidemiology and pathophysiology of mitral valve prolapse: new insights into disease progression, genetics, and molecular basis. Circulation. 2014;129:2158–70. 10.1161/CIRCULATIONAHA.113.006702 24867995 PMC4052751

[18] Beighton P, de Paepe A, Danks D, Finidori G, Gedde-Dahl T, Goodman R, et al International nosology of heritable disorders of connective tissue, Berlin, 1986. Am J Med Genet. 1988;29:581–94. 10.1002/ajmg.1320290316 3287925

[19] Grahame R. Heritable disorders of connective tissue. Baillieres Best Pract Res Clin Rheumatol. 2000;14:345–61. 10.1053/berh.1999.0069 10925749

[20] Tocchioni F, Ghionzoli M, Messineo A, Romagnoli P. Pectus excavatum and heritable disorders of the connective tissue. Pediatr Rep. 2013;5:e15 10.4081/pr.2013.e15 24198927 PMC3812532

[21] Ng CM, Cheng A, Myers LA, Martinez-Murillo F, Jie C, Bedja D, et al TGF-beta-dependent pathogenesis of mitral valve prolapse in a mouse model of Marfan syndrome. J Clin Invest. 2004;114:1586–92. 10.1172/JCI22715 15546004 PMC529498

[22] Creswick HA, Stacey MW, Kelly RE Jr., Gustin T, Nuss D, Harvey H, et al Family study of the inheritance of pectus excavatum. J Pediatr Surg. 2006;41:1699–703. 10.1016/j.jpedsurg.2006.05.071 17011272

[23] Oezcan S, Attenhofer Jost CH, Pfyffer M, Kellenberger C, Jenni R, Binggeli C, et al Pectus excavatum: echocardiography and cardiac MRI reveal frequent pericardial effusion and right-sided heart anomalies. Eur Heart J Cardiovasc Imaging. 2012;13:673–9. 10.1093/ehjci/jer284 22298154

