# Supplementary material for: Characteristics of mitral valve leaflet length in patients with pectus excavatum: A single center cross-sectional study
Source: PLoS One. 2019 Feb 11;14(2):e0212165. doi: 10.1371/journal.pone.0212165 (PMC6370242; doi:10.1371/journal.pone.0212165)
Supplement: S1 Table — Values are presented as ± standard deviation or n (%). PE indicates pectus excavatum. a n = 43. (DOCX) [file pone.0212165.s001.docx]

|  | Normal controls  (n = 46) | PE  (n = 35) | p value |
| --- | --- | --- | --- |
| Patient < 16-year old, n (%) | 4 (8.7) | 19 (54.3) | <0.0001 |
| Age, years | 54.8 ± 19.8 | 19.9 ± 14.7 | <0.0001 |
| Males, n(%) | 29 (63.0) | 23 (65.7) | 0.8038 |
| Height, m | 1.63 ± 0.09 ^a^ | 1.52 ± 0.25 | 0.0065 |
| Body weight, kg | 59.1 ± 14.2 ^a^ | 42.9 ± 18.4 | <0.0001 |
| Body surface area, m^2^ | 1.63 ± 0.22 ^a^ | 1.35 ± 0.40 | 0.0002 |
| Mitral valve prolapse | 0 (0.0) | 1 (2.9) | 0.2487 |

^a^ n =43.
