# Supplementary material for: Characteristics of mitral valve leaflet length in patients with pectus excavatum: A single center cross-sectional study
Source: PLoS One. 2019 Feb 11;14(2):e0212165. doi: 10.1371/journal.pone.0212165 (PMC6370242; doi:10.1371/journal.pone.0212165)
Supplement: S2 Table — ASD indicates atrial septal defect. TR indicates tricuspid regurgitation. AR indicates aortic regurgitation. MVP indicates mitral valve prolapse. (DOCX) [file pone.0212165.s002.docx]

| Patient |  | cardiovascular complications | |
| --- | --- | --- | --- |
| #1 | 3-year-old, male | Kawasaki disease |  |
| #2 | 11-year-old, male | ASD |  |
| #3 | 12-year-old, female | Mild TR (floppy valve) |  |
| #4 | 15-year-old, male | Aortic bicuspid valve | Mild AR |
| #5 | 19-year-old, female | MVP (anterior leaflet) |  |
| #6 | 26-year-old, male | Mild TR (floppy valve) |  |
